# The Relationship Between Posttraumatic Stress Disorder Symptoms and Posttraumatic Growth Among HIV-Infected Men Who Have Sex With Men in Beijing, China: The Mediating Roles of Coping Strategies

**DOI:** 10.3389/fpsyg.2018.01787

**Published:** 2018-09-27

**Authors:** Zhi Ye, Lihua Chen, Danhua Lin

**Affiliations:** ^1^Institute of Developmental Psychology, Beijing Normal University, Beijing, China; ^2^Faculty of Psychology, Beijing Normal University, Beijing, China

**Keywords:** HIV/AIDS, men who have sex with men, coping strategy, posttraumatic growth, posttraumatic stress disorder symptoms, China

## Abstract

The traumatic experience of contracting and living with HIV/AIDS may produce a myriad of mental health problems, especially posttraumatic stress disorder (PTSD) symptoms, and conversely, bring posttraumatic growth (PTG), that is, positive changes resulting from a struggle with trauma. The growing body of research into the relationship between PTSD symptoms and PTG has produced mixed results. In addition, some research has suggested that psychosocial and cognitive factors may mediate the development of PTG after trauma exposure. Specifically, individuals experience fewer psychological symptoms and better mental health when adaptive coping strategies align with stressors; however, little research is available on the relationship and the mediating effect of coping strategies on the link of PTSD symptoms and PTG among HIV-infected men who have sex with men (MSM) in China. The aims of the current study were to investigate the relationship between PTSD symptoms and PTG as well as the potential mediating effects of coping strategies through which PTSD symptoms contributes to PTG among this vulnerable population. One hundred and forty HIV-positive MSM were recruited from the Beijing Center for Disease Prevention and Control and were asked to complete a battery of self-administered questionnaires, covering sociodemographic and HIV-related characteristics, coping strategies (i.e., problem-solving, seeking social support, self-blame, and wishful thinking), PTSD symptoms, and PTG. Results showed that, after controlling for sociodemographic and HIV-related variables, a negative linear relationship was found between PTSD symptoms and PTG. In addition, problem-solving and self-blame played significant mediating roles in the association between PTSD symptoms and PTG. The mediating effects of seeking social support and engaging in wishful thinking on the PTSD symptoms and PTD link were, however, non-significant. The present study contributes to an understanding of the association between PTSD symptoms and PTG and underscores the mediators through which individuals gain growth from traumatic experience in the context of HIV infection in Beijing, China. Given these findings, the future efforts at psychological intervention should differentiate and target various types of coping strategies, especially focusing on enhancing problem-solving skills and decreasing self-blame, in response to the promotion of positive growth among HIV-infected MSM.

## Introduction

The prevalence of HIV among men who have sex with men (MSM) drew relatively little attention until recent decades in China (Wu et al., 2007; Song et al., 2011). Although HIV epidemics have stabilized or fallen among other subgroups (e.g., female sex workers, drug users) under the supervision of the government, homosexual transmission has become the driving force of HIV infection among people living with HIV/AIDS (PLWHA) in China, especially in metropolises like Beijing (UNAIDS, 2015; Choi et al., 2016). In particular, HIV transmission among Chinese MSM increased from 2.50% in 2006 to 25.80% in 2014 (Zhang L. et al., 2013; Choi et al., 2016). According to the national AIDS epidemic report, the annual rate of newly reported HIV-infected MSM cases was approximately 24.4% in 2017 (NCAIDS et al., 2017). HIV-infected MSM have emerged as a high-risk group in China.

Living with HIV is not an acute, singular stressful experience, but a series of unfolding threats by facing various mental and physical challenges, for instance, a lack of supportive social network, high public stigma, exhausting healthcare services, poor quality of life, economic difficulties and decreases of health condition (Guo et al., 2011; Sherr et al., 2011; Sikkema et al., 2013; Li et al., 2016; Lau et al., 2018). In addition, the predominant homophobia in Chinese society combined with misconceptions about HIV has marginalized the Chinese MSM population both culturally and socially, making them more likely to face discrimination (Guo et al., 2011; Ye et al., 2015). Fear of stigmatization and a sense of shame have isolated them from the dominant society (Liu and Choi, 2006; Neilands et al., 2008), resulting in vulnerability to mental health problems, especially posttraumatic stress disorder (PTSD) symptoms (Yu et al., 2017). Previous research under Chinese social context has shown that the prevalence of PTSD among HIV-infected population was approximately 37% (Wang et al., 2015), much higher than in the general population (∼0.3%) (Xi et al., 2017). What’s worse, considerable evidence has shown that PTSD covaries with other types of emotional distress (e.g., depression and anxiety), physical dysfunction, and poor medication adherence (Cadell et al., 2003; Kamen et al., 2012).

Although those living with HIV may develop chronic psychological impairment, they may, nevertheless, also experience positive changes in multiple dimensions, including perception of self, sense of relationship with others, and philosophy of life (Tedeschi and Calhoun, 1995), a phenomenon conceptualized as posttraumatic growth (PTG) (Calhoun and Tedeschi, 1999; Helgeson et al., 2006). Previous research has shown that 59% to 83% of HIV patients reported feelings of PTG after an HIV diagnosis (Siegel and Schrimshaw, 2000; Milam, 2006). In addition, the increase in the levels of PTG were associated with not only the reduction of symptomatology but also the improvement of well-being, optimism, and overall adaptation to HIV (Helgeson et al., 2006; Sawyer et al., 2010).

Previous research has shown that PTSD and PTG often coexist (Tedeschi and Calhoun, 2004); however, mixed results were found in terms of the relationship between these two constructs. Accordingly, four theoretically possible relationships exist with PTSD and PTG (Levine et al., 2008; Wu et al., 2018). For instance, some findings showed that PTSD symptoms would aggravate the traumatic experience and damage one’s psychological and physical function, further decreasing the levels of PTG (Hall et al., 2010; Yi and Kim, 2014). In contrast, some studies suggested a possible positive linear relationship between PTSD and PTG because the latter is an outcome of struggling with the former, which may lead to change and rebuild the individual’s value system (Cadell et al., 2003; Calhoun and Tedeschi, 2006; Taku et al., 2008; Tian et al., 2016). Some researchers have also suggested that PTSD symptoms and PTG may not be related but coexist independently (Cordova et al., 2007; Grubaugh and Resick, 2007); moreover, additional studies have shown a curvilinear relationship with an inverted “U” curve between the two constructs, whereby intermediate levels of PTSD were associated with optimal levels of PTG (Butler et al., 2005; Levine et al., 2008; Kleim and Ehlers, 2009). Although researchers have examined the relationship between PTSD and PTG among populations surviving diverse traumatic contexts, limited studies have been conducted following HIV/AIDS exposure. One of the aims of the present study was, therefore, to examine the relationship between PTSD and PTG among HIV-infected MSM in Beijing, China.

In addition, a better understanding of the pathways through which PTSD links to PTG may allow for tailored psychological behavioral interventions designed to promote positive changes among individuals exposed to HIV-related stress. PTG is not the direct outcome of trauma, conversely, it is suggested as a consequence of individuals’ struggle to deal with traumatic symptomatology through the strength that comes from suffering, existential reevaluation, and psychological preparedness (Tedeschi and Calhoun, 2004). Coping strategies may, therefore, play crucial roles in the development of positive changes after posttraumatic stress symptoms (Marotta-Walters et al., 2015; Dekel et al., 2016). Generally, coping strategies can be divided into two types on the basis of function, named emotion-focused coping and problem-focused coping (Folkman et al., 1986). For individuals who were diagnosed of HIV/AIDS, managing intense emotion reactions or altering the realistic problems is particularly necessary to decline PTSD symptoms (Chernoff, 2007). In turn, people would recover from traumatic experience and develop positive changes through emotional self-control and problem solving (Folkman and Lazarus, 1980; Folkman et al., 1986). Numerous researchers have explored the conflicting effects of various types of coping strategies on mental health outcomes (Sikkema et al., 2007, 2013). Findings indicated that for individuals who exposed to traumatic experience and developed PTSD symptoms, coping strategies oriented away from reactions to stressors, such as denial, wishful thinking, and self-blame, were closely associated with more dissatisfaction of the life and hinder the probability of growth after trauma (Tsay et al., 2001; Dörfel et al., 2008). On the contrary, coping strategies concentrating on solving the problems or reducing stress, such as seeking social support and collecting more information to tackle problems, may help individuals to rebuilding the meanings of life and integrating the traumatic experience with existing cognitive schemas about self and the world, which may furtherly promote the levels of PTG (Luszczynska et al., 2005; Prati and Pietrantoni, 2009; Strasshofer et al., 2017). In particular, this has also been shown in empirical studies targeting the PLWHA population from diverse societies to highlight the cross-culturally valid associations between HIV-related PTSD symptoms, distinct effects of coping strategies and positive growth after trauma (Kraaij et al., 2008; Breet et al., 2014; Rzeszutek et al., 2017; Ye et al., 2018). Nevertheless, the potential mediating effects of different coping strategies on the relationship between PTSD and PTG remain unclear. Especially, rejection of those with HIV/AIDS by the public and the stigma associated with homosexuality in China’s society may have direct influence on ways to cope with HIV-related stress and emotional reactions in this vulnerable population (Choi et al., 2017); therefore, the distinct mediating effects of coping strategies may vary in accordance with its specific types.

Consequently, the current study was designed for two aims. The first was to explore the relationship between PTSD and PTG, specifically, whether the relationship is linear or curvilinear. The second was to investigate how different types of coping strategies (i.e., problem-solving, seeking social support, self-blame and wishful thinking) account for the mechanism underlying the relationship between PTSD and PTG among Chinese HIV-infected MSM.

## Materials and Methods

### Study Design and Participants

This cross-sectional study was carried out at the Beijing Center for Disease Prevention and Control (CDC) between May and July 2013. Previous research emphasized that special attention should be paid to newly diagnose cases because of not only their adjustment difficulties to new disease identity, but also the lasting effect of initial phase of health status on mental and physical health outcomes (Li et al., 2014; Lau et al., 2018). Additionally, newly diagnose of HIV is also a critical time for the development of PTSD and PTG and it may take time for PTG to emerge in the aftermath of HIV-related events (Yu et al., 2017; Lau et al., 2018). Consequently, the eligible participants for this study were (1) males; (2) over 18 years of age; (3) those who had reported ever engaging in same-gender sex; and (4) those who received their first notification of HIV infection within 3 years prior to the study. With the help of healthcare workers at Beijing CDC, all eligible clients were told about the aim and content of the study; and those who were interested in it were invited to join and complete the questionnaire. A total of 140 participants joined the study, and all of them provided written informed consent and self-administered the assessment protocol. Participants received incentives of 100 CNY (1 USD = 6.30 CNY) to compensate for time spent in completing assessments. This study obtained approval from the Institutional Review Board of Beijing Normal University.

### Measures

#### Demographic and HIV-Related Characteristics

The participants provided their gender, age, educational level (0 = secondary or lower, 1 = tertiary or higher), employment status (0 = Unemployed/ Part-time employment, 1 = full-time employment), marital status (0 = single, 1 = married), and monthly income (1 = 0 – 999 Yuan, 2 = 1000 – 1999 Yuan, 3 = 2000 – 2999 Yuan, 4 = 3000 – 3999 Yuan, 5 = 4000 – 4999 Yuan, 6 = 5000 Yuan and more) as well as the amount of time since the HIV diagnosis and treatment with antiretroviral therapy (ART) (0 = no, 1 = yes).

#### Posttraumatic Stress Disorder Symptoms

Based on theoretical notions and operationalization of PTSD symptoms, the Impact of Events Scale (IES) (Horowitz et al., 1979) was used to assess posttraumatic stress after HIV diagnosis. The scale consists of 15 items, including seven intrusion items and eight avoidance items. Participants rated the frequency of PTSD experience and symptoms during the previous 7 days on a 4-point scale, ranging from 1 (*not at all*) to 4 (*often*). The IES is a widely used, psychometrically reliable, and valid self-report measure of PTSD symptoms among HIV-infected populations (Chernoff, 2007; Sikkema et al., 2007). The internal consistency for intrusion subscale and avoidance subscale in the original study was 0.78 and 0.82 (Horowitz et al., 1979), respectively. The internal consistency for intrusion and avoidance subscales in the current research was 0.90 and 0.83, respectively.

#### Coping Strategies

The Ways of Coping Checklist-Revised (WCC) (Vitaliano et al., 1985) was used to assess participants’ behavioral and cognitive responses to stressful situations. The WCC is a 41-item scale with five subscales that represent five types of coping strategies, including problem-solving, seeking social support, self-blame, wishful thinking, and avoidance. Participants rated the use of coping strategies in the face of stressful situations on a 5-point scale, ranging from 0 (*strongly disagree*) to 4 (*strongly agree*). In the current study, we excluded the avoidance subscale because of its high conceptual overlap and statistical collinearity with the avoidance subscale of PTSD. The reliability and validity of the scale were great and the internal consistencies of the problem-solving, seeking social support, self-blame and wishful thinking subscales in the original study were 0.88, 0.85, 0.75, and 0.78 (Vitaliano et al., 1985). The internal consistencies for the four subscales in the current study was 0.91, 0.76, 0.75, and 0.79, respectively.

#### Posttraumatic Growth

The 21-item Posttraumatic Growth Inventory (PTGI) was used to assess the features of positive changes after HIV-related experiences (Tedeschi and Calhoun, 1996). The scale contains five subscales: relating to others, personal strength, new possibilities, appreciation of life, and spiritual change. Previous studies had shown that asking spiritual questions among Chinese participants is inappropriate (Yu et al., 2010; Zhang Y.J. et al., 2013), therefore, the subscale on spiritual change with two items was excluded from the assessment protocol. Participants rated their growth after HIV diagnosis on a 4-point scale, ranging from 1 (*not at all*) to 4 (*very much*). The reliability of the scale was great in the original study with the internal consistencies for relating to others, personal strength, new possibilities and appreciation of life subscales were 0.85, 0.72, 0.84, and 0.67 (Tedeschi and Calhoun, 1996). In this study, internal consistencies for subscales were 0.82, 0.83, 0.77, and 0.76, respectively.

### Statistical Analysis

First, descriptive statistics, including means, standard deviations, and bivariate correlations of demographic and HIV-related characteristics as well as all the variables were calculated using SPSS 22.0. Then, the linear and curvilinear effects of PTSD on PTG were evaluated using Mplus 7.1 (Muthén and Muthén, 2012). Finally, multiple mediation analysis with four mediators was conducted using Structural Equational Modeling (SEM). In the mediation model, age, educational level, employment statement, marital status, monthly income, duration of HIV diagnosis and the status of antiretroviral treatment (ART) were controlled for as potential confounders. Maximum likelihood estimation with bootstrapped (5,000 samples) asymmetric confidence intervals (CIs) was used for testing indirect effects in the model. The indirect effects are considered statistically significant when the confidence intervals do not include zero. To enhance the interpretability of results, several key indicators were used to test the model fit, including the root mean square error of approximation (RMSEA) (0.08 or below reflecting an acceptable fit), standardized root mean square residual (SRMR) (0.05 or below indicating excellent fit), comparative fit index (CFI) (0.90 or above indicating an acceptable fit), and the Tucker-Lewis index (TLI) (0.90 or above indicating an acceptable fit).

## Results

### Descriptive Statistics of Demographic and HIV-Related Characteristics

Demographic and HIV-related characteristics of the participants appear in **Table 1**. Among the 140 participants, the majority of them had obtained tertiary education or higher (115, 82.1%) and were single (138, 98.5%). The average age was 26.6 (*SD* = 3.3, range: 19–36 years). The mean duration since their HIV diagnosis was 4.7 months (*SD* = 5.2, range: 1–36 months, **Table 1**). The bivariate correlation matrix of PTSD symptoms, coping strategies, and PTG appear in **Table 2**.

**Table 1:** Demographic and HIV-related characteristics of the participants (*N* = 140).

	*M* ±*SD* or *n* (%)
Age	26.6 ± 3.3(19–36)
**Educational level**	
Secondary or lower	25 (17.9)
Tertiary or higher	115 (82.1)
**Employment**	
Unemployed/Part-time employment	37 (26.5)
Full-time employment	96 (68.6)
Unknown	7 (5.0)
**Marital status**	
Single	138 (98.5)
Married	1 (0.7)
Unknown	1 (0.7)
**Monthly income (CNY^a^)**	
0–999	11 (7.9)
1000–1999	7 (5.0)
2000–2999	22 (15.7)
3000–3999	27 (19.3)
4000–4999	23 (16.4)
≥5000	48 (34.3)
Unknown	2 (1.4)
Duration of HIV diagnosis (months)	4.7 ± 5.2(1–36)
**Receiving antiretroviral treatment**	
Yes	121 (86.4)
No	19 (13.6)


*^a^1 USD = 6.30 CNY.*

**Table 2:** Descriptive statistics and correlation coefficients among variables.

	*M* ±*SD*	1	2	3	4	5	6	7	8	9	10
**Posttraumatic stress disorder (PTSD)**		
(1) Avoidance	18.44 ± 4.63	1	0.64***	–0.28***	–0.22***	0.35***	0.24***	–0.16	–0.15	–0.19*	–0.09
(2) Intrusion	18.36 ± 5.23		1	–0.24***	–0.14	0.46***	0.33***	–0.13	–0.18*	–0.28***	–0.04
**Coping strategies**		
(3) Problem-solving	2.54 ± 0.59			1	0.62***	–0.07	0.20*	0.56***	0.60***	0.59***	0.47***
(4) Seeking social support	2.35 ± 0.70				1	0.05	0.23***	0.55***	0.48***	0.46***	0.36***
(5) Self-blame	2.10 ± 0.85					1	0.55***	–0.17*	–0.14	–0.26***	–0.13
(6) Wishful thinking	2.49 ± 0.67						1	0.07	0.02	–0.02	0.12
**Posttraumatic growth (PTG)**		
(7) Relating to others	19.79 ± 4.57							1	0.80***	0.75***	0.62***
(8) New possibilities	14.15 ± 3.60								1	0.87***	0.70***
(9) Personal strength	11.84 ± 2.84									1	0.63***
(10) Appreciation of life	9.41 ± 2.10										1


*^∗^p < 0.05; ^∗∗^p < 0.01; ^∗∗∗^p < 0.001.*

### The Relationship Between PTSD and PTG

After controlling for demographic and HIV-related characteristics, the relationship between PTSD symptoms and PTG was tested with Mplus 7.1. Initially, the latent construct of PTG was regressed onto the latent construct of PTSD symptoms linearly and the results indicated a negative association between PTSD and PTG (*B* = -0.28, *SE* = 0.12, *p* < 0.05). Then, PTSD was squared to create a quadratic term and was added into the model. The results showed that the quadratic effect of PTSD on PTG was not statistically significant (*B* = -0.05, *SE* = 0.05, *p* = 0.30), whereas the linear association remained significant (*B* = -0.29, *SE* = 0.14, *p* < 0.05). Taken together, the findings revealed that the association between PTSD and PTG was negative linear in the current sample, suggesting that HIV-infected MSM who reported fewer PTSD symptoms might have higher levels of PTG.

### Mediating Effects of Coping Strategies on the Association of PTSD and PTG

**FIGURE 1:**
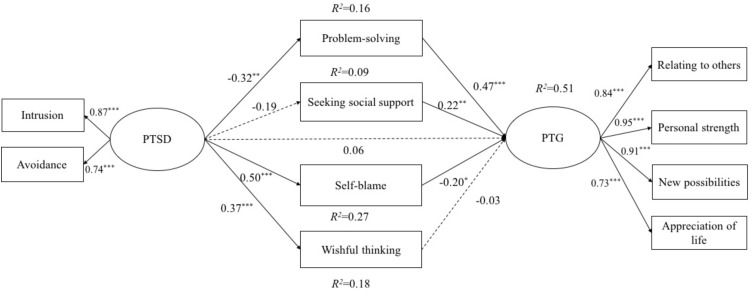
The path analysis results of the mediation model linking PTSD to PTG. Significant pathways are represented with solid lines, and nonsignificant pathways are represented with dashed lines. Covariates including demographic and HIV-related characteristics were controlled in the model but not presented in this figure. PTSD, posttraumatic stress disorder; PTG, posttraumatic growth. *^∗^p* < 0.05*; ^∗∗^p* < 0.01*; ^∗∗∗^p* < 0.001.

A multiple mediation model was established to test the mediating effects of four types of coping strategies (see **Figure 1**). After controlling for the demographic and HIV-related characteristics, the mediation model provided an excellent fit with the data: *χ^2^*/*df* = 1.42, TLI = 0.95, CFI = 0.98, RMSEA = 0.06, and SRMR = 0.04. Significant indirect effects were shown from PTSD through problem-solving (indirect effect = -0.15, 95% CI = [-0.28, -0.03]) and self-blame (indirect effect = -0.10, 95% CI = [-0.19, -0.01]) on PTG. We calculated the ratio of the indirect effect for specific mediator to the total effect (Preacher and Kelley, 2011) and found that problem-solving coping strategy mediated approximately 63% of the total effect of PTSD on PTG as well as self-blame coping strategy mediated approximately 42% of the total effect of PTSD on PTG. Specifically, lower levels of PTSD might motivate individuals to focus on solving the problems they face (*β* = -0.32, *p* < 0.01) and then result in higher levels of PTG (*β* = 0.47, *p* < 0.001); conversely, higher levels of PTSD were associated with higher frequency of self-blame (*β* = 0.50, *p* < 0.001), which in turn would decrease the level of PTG (*β* = -0.20, *p* < 0.05). Regarding to the mediating roles of seeking social support and wishful thinking, the results from bootstrapping revealed that neither of them were significant mediators on the link between PTSD and PTG (see **Table 3**). Besides, the direct effect of PTSD on PTG was no longer significant (*β* = 0.06, *p* = 0.57, direct effect = 0.06, 95% CI = [-0.14, 0.26]).

**Table 3:** Direct and indirect effects between all the variables from bootstrap analysis.

Pathways	Estimated effect	95% CI	Effect size
**Direct effect**	
PTSD→PTG	0.06	[–0.14, 0.26]	
**Indirect effect**	
PTSD→ Problem-solving →PTG	–0.15^∗^	[–0.28, -0.03]	0.63
PTSD→ Seeking social support →PTG	–0.04	[–0.10, 0.01]	0.17
PTSD→ Self-blame →PTG	–0.10^∗^	[–0.18, -0.01]	0.42
PTSD→ Wishful thinking →PTG	–0.01	[–0.07, 0.05]	0.04


*PTSD, posttraumatic stress disorder; PTG, posttraumatic growth. *^∗^p* < 0.05.*

## Discussion

The current study was among the first to investigate the relationship between PTSD symptoms and PTG, and the potential mediating effects of four types of coping strategies on the relationship of two constructs using a sample of HIV-infected MSM in Beijing, China. The findings suggest that, contrary to the results of some previous research, a negative linear relationship exists between PTSD symptoms and PTG. Moreover, in terms of the mediating effects of the four types of coping strategies, problem-solving and self-blame played opposite mediating roles in the relationship of PTSD symptoms and PTG, but seeking social support and wishful thinking made no contribution to the understanding of the relationship between PTSD symptoms and PTG. The findings extended the existing research, showing the potential relationship between PTSD symptoms and PTG among HIV-infected population and addressing a gap to explore the pathway through which PTSD symptoms may predict PTG.

Our study adds to the literature because it targets the young MSM population, considered the most-at-risk group of HIV infection in China (Guo et al., 2011). Predominant homophobia and misconceptions about HIV in Chinese society made this subgroup more likely to stay away from the public (Choi et al., 2004; Wong et al., 2006). What’s worse, although the government has made efforts to combat the HIV/AIDS epidemic, adequate services and protection targeting MSM are still limited (Choi et al., 2002; Guo et al., 2011). Consequently, discussion about mental health status is urgently needed to provide necessary psychological supports for this vulnerable population (Li et al., 2014).

Contrary to the mixed results found in other traumatized populations (Cadell et al., 2003; Butler et al., 2005; Kleim and Ehlers, 2009; Hall et al., 2010; Strasshofer et al., 2017), a negative linear relationship between PTSD and PTG was found in the study, showing that greater levels of PTSD symptoms are related to lower levels of PTG in a linear way. The findings contribute to expanding the previous theoretical knowledge to a culturally and socially marginalized population, showing that PTSD symptoms and PTG co-occur simultaneously and posttraumatic stresses may disturb individuals’ attempts to reconstruct their values in relations to life and the world, which in turn, may disrupt the process of personal growth directly (Johnson et al., 2007; Yi and Kim, 2014). Notably, the relationship found in the current study between PTSD symptoms and PTG is specific to the HIV-infected MSM population. HIV/AIDS trauma may be unique in terms of its nature as a chronic, life-threatening disease like cancer as well as its destruction to lives and social interaction (Johnson et al., 2007). Especially in China, HIV-infected MSM have been found to experience multiple stressors in daily life, including homosexuality- and HIV-related discrimination, economic burdens, interpersonal relationship problems, emotional distresses, and isolation from society, the negative influence of which can last for a long time (Guo et al., 2011; Pineles et al., 2013; Ye et al., 2015, 2018; Choi et al., 2016). Consequently, the differences in characteristics between HIV-infected MSM and survivors of other kinds of trauma might explain the negative linear relationship between PTSD symptoms and PTG in this study.

In addition, we extended existing research by demonstrating that coping strategies acted as pathways through which PTSD symptoms predict PTG. Our findings indicated the distinct mediating effects of different types of coping strategies in the link of PTSD symptoms and PTG. Particularly, on one hand, lower PTSD symptoms may increase the level of problem-solving coping strategy, and in turn, promote the levels of PTG. Previous research showed that among the multiple cognitive and emotional components, positive reappraisal of the trauma-related experience and focusing on how to deal with the stressful situation are specifically effective and useful for later personal growth (Tedeschi and Mcnally, 2011; Rzeszutek et al., 2017). Lower levels of PTSD symptoms may motivate clients to focus on how to solve the problems in daily life (e.g., economic stress, health maintenance and interpersonal relationships) rather than managing the emotional reaction to the traumatic events, which furtherly enhance the attempts at mastery of HIV-related stressful events and promote self-efficacy and positive changes (Luszczynska et al., 2005). On the other hand, higher levels of PTSD symptoms may result in higher level of self-blame and decrease the levels of PTG. Previous research indicated that the hallmark symptom of PTSD is emotional distress in response to severe disease- and sexual orientation-related life stress from jobs and interpersonal relationships (Pineles et al., 2013; Li et al., 2016). Individuals’ uncontrollable memories of the trauma, or more avoidant reactions toward HIV experience, may bring more frequent inner guilty and blame to the unsafe sexual experience and carelessness of themselves, which further hinder the development of positive changes (Feldner et al., 2007). Although previous research suggested that self-blame could be adaptive to help individuals regain belief in their capacities to control events in future lives to some extent (Janoff-Bulman, 2006); however, HIV/AIDS -related stress is life-threatening and somewhat uncontrollable objectively (Johnson et al., 2007). Higher levels of PTSD symptoms may remind clients of past traumatic experiences frequently and increase maladaptive self-blame, which furtherly decrease the sense of future control, and in turn, decrease growth (O’Neill and Kerig, 2000; Frazier et al., 2001).

Notably, neither engaging in wishful thinking nor seeking social support played the role of mediator in the relationship between PTSD and PTG. On the one hand, although PTSD was positively related to wishful thinking, the predictive effect of wishful thinking on PTG was not significant. That may be because in the face of HIV-related traumatic experience, people tend to respond with a distorted positive perception of themselves, an exaggerated sense of personal control, and unrealistic optimism to decrease the severe symptoms of PTSD symptoms (Davis and McKearney, 2003; Sikkema et al., 2013); but doing so may not be helpful in overcoming the psychological barriers, reframing new world assumptions, and taking adaptive actions to promote PTG and positive outcomes (Sherr et al., 2011; Garrido-Hernansaiz et al., 2017). On the other hand, we found a positive link between seeking social support and PTG, whereas, non-significant association was found between PTSD and seeking social support. Theoretically, social support is considered as a key resource for understanding positive changes in the aftermath of trauma (Schaefer and Moos, 1998; Tedeschi and Calhoun, 2004). Previous research has consolidated the positive effect of seeking social support on improving social resources by providing sympathy and reducing feelings of isolation and loneliness and further reframing positive, new world assumptions, which lead to PTG (Cieslak et al., 2009; Prati and Pietrantoni, 2009; Roepke, 2015; Rzeszutek et al., 2017). Nevertheless, the findings in other populations indicate that PTSD symptoms would bring greater cognitive social avoidance and lower availability of secure interpersonal relationships, which may prevent individuals from seeking social support from others to some extent (Tsai et al., 2012). Moreover, as for HIV-infected MSM in China, the sense of shame and fear may prevent them from disclosing traumatic experiences and emotion distress to family members or friends (Choi et al., 2004; Wong et al., 2006); therefore, seeking social support may not be an efficient coping strategy for clients to decrease PTSD symptoms.

Some potential limitations of this study should be considered when interpreting our findings. First, the sample was restricted to voluntary participants in CDC and the sample size was small; therefore, the results should be interpreted with caution and may not be generalizable to the diverse HIV-infected MSM population in China. Given the hidden nature of the MSM population in China, future researchers should design an appropriate combination of multiple sampling methods and larger samples to access this population from diverse backgrounds to improve the representativeness of the studies. Second, all variables in the current study were measured using self-report scales, which may lead to some potential bias (e.g., social desirability response and error in recall) in estimating associations. Future research might include biological and behavioral indicators or observer reports, for example, participants’ detailed medication information (e.g., the specific prescribed HIV infection medicine), CD4 counts or health conditions. Last but not least, the nature of cross-sectional design limits the interpretation of causality among PTSD, coping strategies and PTG. Future directions should include longitudinal design to examine how the relationship from PTSD to PTG through four types of coping strategies unfolds over time.

Despite these limitations, the current study provides insights and important implications for future research, interventions, and health education. Exploring the relationship between PTSD and PTG can help researchers to broaden the understanding of HIV-infected populations’ psychological adaptation after traumatic experience related to HIV/AIDS. Additionally, the investigation of underlying mechanisms of the link between PTSD symptoms and PTG have further important implications for implementation efforts to promote positive mental health and physical health among this vulnerable population. Particularly, the findings highlighting the distinct mediating roles of coping strategies, throw light on new research directions to understand the valuable effects of coping strategies in HIV-infected populations. The current study indicated that culturally relevant integrated intervention programs should target at developing adaptive coping skills and reducing maladaptive coping skills to cultivate PTG and its related positive outcomes among HIV-infected MSM, other types of PLWHA, and even other disease groups.

## Author Contributions

ZY wrote the first draft of the manuscript and assisted in study design, data collection, and data analyses. LC drafted the work and revised it critically for important intellectual content. DL was the principal investigator of the study and led the study. All of the authors participated in the final approval of the version to be published and agreed to be accountable for all aspects of the work.

## Conflict of Interest Statement

The authors declare that the research was conducted in the absence of any commercial or financial relationships that could be construed as a potential conflict of interest.
